# A systematic review of the use and reporting of evaluation frameworks within evaluations of physical activity interventions

**DOI:** 10.1186/s12966-020-01013-7

**Published:** 2020-08-24

**Authors:** Judith F. Fynn, Wendy Hardeman, Karen Milton, Joseph Murphy, Andy Jones

**Affiliations:** 1 UKCRC Centre for Diet and Activity Research (CEDAR) and Norwich Medical School, University of East Anglia, Norwich, UK; 2 School of Health Sciences, University of East Anglia, Norwich, UK; 3 Norwich Medical School, University of East Anglia, Norwich, UK; 4 Physical Activity for Health Research Cluster, Physical Education and Sport Sciences Department, University of Limerick, Limerick, Ireland

**Keywords:** Evaluation framework, Physical activity, Systematic review, Intervention

## Abstract

**Background:**

Evaluation of physical activity interventions is vital to inform, and justify, evidence-based policy and practice to support population-wide changes in physical activity. Several evaluation frameworks and guidance documents have been developed to facilitate the evaluation and reporting of evaluation studies in public health. However, there is a lack of evidence about whether frameworks are being used to guide evaluation. There continues to be claims of poor and inconsistent reporting in evaluation studies. The aim of this review was to assess the use of evaluation frameworks and the quality of reporting of how they were applied within evaluation studies of physical activity interventions.

**Objectives:**

1. To identify whether evaluation frameworks are reported to have been used within evaluation studies of physical activity interventions, and which frameworks have been used.
2. To appraise the quality of reporting with regards to how evaluation frameworks have been used.

**Method:**

We developed a checklist of indicators to enable a critical appraisal of the use and reporting of different evaluation frameworks in evaluation studies. We conducted a systematic search and review of evaluation studies published between 2015 and the date of the search to appraise the use and reporting of evaluation frameworks. A narrative synthesis is provided.

**Results:**

The review identified 292 evaluation studies of physical activity interventions, only 69 (23%) of these mentioned using an evaluation framework, and only 16 different frameworks were referred to. There was variation in the quality of reporting of framework use. 51 (74%) studies were identified as being explicitly based on the stated framework, however only 26 (38%) provided detailed descriptions consistently across all the checklist indicators. Details of adaptations and limitations in how frameworks were applied were less frequently reported. The review also highlighted variability in the reporting of intervention components. More consistent and precise reporting of framework and intervention components is needed.

**Conclusion:**

Evaluation frameworks can facilitate a more systematic evaluation report and we argue their limited use suggests missed opportunities to apply frameworks to guide evaluation and reporting in evaluation studies. Variability in the quality of reporting of framework use limits the comparability and transferability of evidence. Where a framework has been used, the checklist of indicators can be employed to facilitate the reporting of an evaluation study and to review the quality of an evaluation report.

## Introduction

Increasing physical activity levels among the population is a public health priority [1–3]. Yet the diversity of individual, environmental and societal influences on physical activity requires interventions that reflect that diversity [3]. This has led to various interventions targeting physical activity behaviour that are delivered to different populations and across many settings by a range of public, private and voluntary providers, many of which are multi-sectoral and multi-component. The complexity and heterogeneity in interventions poses challenges to understanding their effectiveness, and to generalising from one intervention to another [4, 5]. Given the high rates of inactivity [6, 7] and the importance of physical activity for health [8], it is vital that we learn from the interventions delivered about what works, for whom, and in what contexts [9].

Over the past 20 years, there has been a growing appreciation of the importance of evaluation to inform evidence-based interventions to support population-wide changes in physical activity and to justify policy and practice [9–11]. Evaluation can be defined as the “systematic examination and assessment of the features of an initiative and its effects, in order to produce information that can be used by those who have an interest in its improvement or effectiveness” [12], p3. Translation from one setting to another, and wider scale adoption of effective interventions, requires both rigorous evaluation and robust reporting of evaluations to build the evidence-base [11, 13].

Several frameworks and guidance documents have been developed to facilitate the evaluation and reporting of intervention studies in public health. In this review the term ‘evaluation framework’ is used to include any structured guidance which facilitates a systematic evaluation of the implementation or outcomes of an intervention. A recent scoping review that we conducted identified 68 evaluation frameworks that could be used to guide evaluation of physical activity interventions [14]. This included frameworks intended to support evaluation of physical activity interventions specifically (e.g. The Standard Evaluation Framework (SEF) for Physical Activity Interventions [15]), as well as frameworks intended to guide development and evaluation of various public health interventions, such as RE-AIM [10], and the Medical Research Council (MRC) guidance on the development and evaluation of complex interventions [16]. We have included more general guidance, such as Logic Models [17], where these provide information or a structure to facilitate a systematic approach to identifying and reporting intervention objectives, activities and outcomes. Several checklists have also been developed to improve the completeness of reporting and quality of intervention descriptions; for example the STROBE Statement for Reporting Observational studies in Epidemiology [18, 19] and the Template for Intervention Description and Replication (TIDieR) [20]. Further, the Behaviour Change Wheel [21] and the Behaviour Change Technique (BCT) Taxonomy V1 [22] provide a framework to facilitate intervention development, that can also be applied to help standardise how the content of behaviour change interventions are specified. Despite the publication of these frameworks and guidance, there is a lack of evidence about whether frameworks are being used to guide evaluation.

There has been continued calls for better evaluation and reporting within public health [23, 24]. In particular, the need for more detailed descriptions of intervention components and contextual factors to help evaluate how, why and in what contexts interventions may be effective, and to allow implementation of good practice [21, 25]. Many of the frameworks and guidance have sought to address this and provide guidance on process evaluation and contextual factors. However, questions remain regarding if and how these frameworks are used within evaluation studies.

Two previous reviews have focused specifically on the use of RE-AIM [26] and the SEF for physical activity interventions [23]. These reviews concluded that the reporting of framework components was inconsistent, and that details related to participants, recruitment and broader effects were particularly poorly reported, despite these being components of the frameworks used. Both reviews also highlighted a need for greater clarity in the reporting of how frameworks have been used. Heterogeneity in the format and guidance provided by frameworks may lead to heterogeneity in the way they are applied. This creates difficulties for those interested in further development of evaluation guidance, and those interested in understanding and comparing the effectiveness of interventions including reviewers of evaluation studies and practitioners or researchers wishing to implement or further develop interventions. This limits the contribution evaluation studies make to the evidence base. Given the extensive number of evaluation frameworks, a better understanding of current practices in the use and reporting of them is needed so that future recommendations related to the use of frameworks and evaluation can be developed appropriately.

The aim of this review was therefore to assess the use of evaluation frameworks and the quality of reporting of how they were used within evaluations of physical activity interventions. The primary objective was to explore whether evaluation frameworks are reported to have been used within evaluation studies of physical activity interventions, and which frameworks have been used. The second objective was to appraise the quality of reporting with regards to how evaluation framework use has been reported. Previous reviews [23, 26] have assessed use of a single evaluation framework against the criteria specified in that framework. To our knowledge, no previous review has developed a set of generic indicators to facilitate the appraisal of the use of multiple evaluation frameworks in reported studies. We therefore developed and applied a set of indicators that would enable a critical appraisal of the use and reporting of different evaluation frameworks in evaluation studies.

## Methods

### Protocol & registration

Search methods and inclusion criteria were specified in advance and registered on PROSPERO (CRD42018089472). We applied the PRISMA statement for reporting items for systematic reviews [27].

### Search strategy

We searched Scopus, CINAHL, and EMBASE for published evaluation studies of physical activity interventions. We used free search terms and MeSH terms relating to evaluation, e.g. program* evaluation, programme effectiveness, process evaluation and outcome evaluation. We also included names of specific evaluation frameworks that we had identified in our scoping review of evaluation frameworks [14], to minimize the risk of missing frameworks that do not include the term evaluation in their title (e.g. RE-AIM). These terms were then combined with terms relating to physical activity behaviours (e.g. physical activity, sport, exercise, sedentary). Table 1 provides the full electronic search strategy for Scopus. The context of this review was to understand current practice and use of frameworks in evaluation studies of physical activity programmes. Therefore, the search was limited to studies published between 2015 and the date of the search (25th March 2019). Only studies published in the English language were included.

**Table 1:** Search strategy applied in CINAHL data base

	Search applied in CINAHL
1	TITLE-ABS-SUBJECT (“program* evaluation”) Published Date: 20150101–20,191,231
2	TITLE-ABS-SUBJECT (“service evaluation”) Published Date: 20150101–20,191,231
3	TITLE-ABS-SUBJECT (“process evaluation”) Published Date: 20150101–20,191,231
4	TITLE-ABS-SUBJECT (“implementation evaluation”) Published Date: 20150101–20,191,231
5	TITLE-ABS-SUBJECT (“program* effectiveness”) Published Date: 20150101–20,191,231
6	TITLE-ABS-SUBJECT (“outcome evaluation”) Published Date: 20150101–20,191,231
7	TITLE-ABS-SUBJECT (“re-aim”) Published Date: 20150101–20,191,231
8	TITLE-ABS-SUBJECT (“standard evaluation framework”) Published Date: 20150101–20,191,231
9	TITLE-ABS-SUBJECT (“intervention mapping”) Published Date: 20150101–20,191,231
10	TITLE-ABS-SUBJECT (“program impact pathway”) Published Date: 20150101–20,191,231
11	TITLE-ABS-SUBJECT (“process evaluation of complex interventions”) Published Date: 20150101–20,191,231
12	TITLE-ABS-SUBJECT (“developing and evaluating complex interventions”) Published Date: 20150101–20,191,231
13	TITLE-ABS-SUBJECT (“framework for program evaluation in public health”) Published Date: 20150101–20,191,231
14	TITLE-ABS-SUBJECT (“logic model”) Published Date: 20150101–20,191,231
15	1 OR 2 OR 3 OR 4 OR 5 OR 6 OR 7 OR 8 OR 9 OR 10 OR 11 OR 12 OR 13 OR 14
16	TITLE-ABS-SUBJECT (“physical activity”) Published Date: 20150101–20,191,231
17	TITLE (exercise) Published Date: 20150101–20,191,231
18	TITLE (MH “exercise”) Published Date: 20150101–20,191,231
19	TITLE-ABS-SUBJECT (sedentary) Published Date: 20150101–20,191,231
20	TITLE-ABS-SUBJECT (sport*) Published Date: 20150101–20,191,231
21	TITLE-ABS-SUBJECT (inactiv*) Published Date: 20150101–20,191,231
22	TITLE-ABS-SUBJECT (fitness) Published Date: 20150101–20,191,231
23	16 OR 17 OR 18 OR 19 OR 20 OR 21 OR 22
24	15 AND 23

All studies identified from the searches were downloaded into the Endnote reference manager and duplicates were removed. Screening of all studies was completed by the lead author. At each stage of the screening process (title, abstract and full paper) a sample of 20 % of studies were checked and validated independently by a second author (JM). Disagreements were resolved through discussion.

### Study selection

Inclusion and exclusion criteria were defined a priori and applied to all papers (see Table 2 for full details). Our interest was in evaluation studies, therefore other articles including conceptual papers, reviews, and research protocols were excluded. To assess the use, and any limitations in the use, of evaluation frameworks across the full range of physical activity interventions we screened the papers to identify studies where increasing physical activity was the stated primary goal, irrespective of whether they reported the use of specified frameworks. We included evaluation studies of any physical activity intervention delivered in any individual, group or population setting (e.g. health care, schools, and geographical areas). We included studies of interventions delivered to the general population as well as to participants diagnosed with a disease (e.g. heart disease, diabetes) or as having one or more disease risk factors (e.g. inactive, obese). We then screened these to identify those studies that had referred to an evaluation framework, and to exclude those that had not mentioned one. We screened the reference lists of the included studies to identify any companion papers, for example, where process and outcome evaluations were reported separately.

**Table 2:** Inclusion and exclusion criteria

Included	Excluded
Published evaluation studies including real-world or service evaluations, randomised control trials, observational and natural experiments, feasibility and pilot studies, outcome and process evaluations, quasi-experimental, pre-post designs, effectiveness and impact studies. All types of evaluations using quantitative and/or qualitative methods will be included, whether they have used specified frameworks or not.	Commentaries or discussion papers, conceptual papers, published extracts, books, editorials, systematic reviews, clinical case-reports, research protocols and reported programme designs.
Reported evaluation studies of programmes that have increasing physical activity as the primary stated goal of the programme, including reduced sitting time or sedentary behaviour.	Reported evaluation studies of programmes that have other health behaviours as the primary stated goal of the programme, e.g. smoking, alcohol, substance abuse, eating disorder behaviours. Reported evaluation studies that state other behavioural outcomes or clinical measures as the primary goal of the programme, e.g. programmes aimed at weight loss, maintaining a healthy weight, prevention or management of diabetes, prevention of stroke or heart attack, improvement of aerobic or cognitive function, reduction of fall,; improvement of physical performance/function through physical activity or exercise.
Evaluations of programmes that align with approaches to behaviour change, i.e. programmes that correspond to any of the nine intervention functions on the Behaviour Change Wheel (education, persuasion, incentivisation, coercion, training, enablement, modelling, environmental restructuring and restrictions) [21].	Evaluations of programmes that do not correspond to any of the nine intervention functions on the Behaviour Change Wheel (education, persuasion, incentivisation, coercion, training, enablement, modelling, environmental restructuring and restrictions).
Studies that referred to one or more evaluation frameworks.	Studies that did not refer to any evaluation framework.

### Data extraction

To address the first objective, we extracted the names of any evaluation frameworks that had been reported as being used in any of the studies. For reporting purposes, we also noted the number of physical activity evaluation studies in which no framework was mentioned. To address the second objective, we extracted data from studies that reported the use of one or more evaluation frameworks. Criteria for data extraction were identified and agreed by all authors a priori*.* Data extraction was completed using a data extraction table.

To assess the context and circumstances in which evaluation frameworks had been used, we extracted data related to study characteristics. So that this review met PRISMA recommendations for the reporting of systematic reviews [27] we used PRISMA guidelines to inform the data we extracted from the studies. In addition we used STROBE for the reporting of observational studies and natural experiments [19], and the TIDieR checklist [20] to guide our data extraction. We extracted data related to study population, intervention setting and components, study design, and process and outcome measures. To help us to characterise the intervention types we extracted data related to the nine intervention functions of the Behaviour Change Wheel, and the activities delivered, where these were explicitly reported. Intervention functions are broad categories to define the general means by which an intervention might change behaviour (e.g. Education, Enablement, and Incentivisation) [21, 28]. Their use in intervention development and reporting is intended to facilitate clearer descriptions of intervention components [21]. This is essential for evaluation and implementation [25]. We applied the nine intervention functions to guide a systematic approach to identify and report study characteristics.

To assess the quality of reporting of the use of the frameworks, we developed a set of data extraction criteria related to how the studies had described a framework and its application. To ensure that we identified a set of indicators that could be applied across any evaluation framework, rather than a specific framework, we used a similar approach to that described by Michie and Prestwich in their coding scheme for assessing the use and reporting of theory in intervention studies [29]. We developed a set of indicators that would allow a systematic examination of how the use of a framework had been reported within each study. Each indicator required a yes/no/not sure response and supporting evidence. We adapted their categories and indicators which aligned closely to our own objectives. For example, Category 1 “Reference to underpinning theory” aligned to our objective to identify any “Reference to an evaluation framework”. Within this category we included four indicators that together assessed the extent to which the framework had been referred to and described to enable us to appraise whether or not the evaluation study was explicitly based on or informed by one or more frameworks. For other items, our indicators were more loosely based on those of Michie and Prestwich. Category 2 and 3 included three indicators to assess the extent to which the methods, data collection and outcomes reported were linked to the specified framework’s components. Category 4 included two indicators to assess the extent to which additional information on how the framework had been used is reported. This last category is important, as there may be good justification for reporting on some rather than all of the components in a framework, or adapting how a framework is applied within a specific evaluation study, but without that information it is difficult to appraise its use and reporting. Any one indicator taken in isolation might seem deficient, so the indicators are best considered together within each category and across the full checklist to provide an overall assessment of how use of a framework has been reported. The criteria were discussed and agreed by all authors. The checklist of categories and indicators is shown in Table 3.

**Table 3:** Categories and indicators for assessing the quality of reporting of the use of evaluation frameworks

Category	Data Extraction Indicators (options for responses)
1. Reference to Framework.	1. Is the framework mentioned even if the study is not explicitly based on it? *Yes/No/Not sure* 2. Does the study refer to 1 or more frameworks? *State number* 3. Is the framework mentioned in the introduction? *Yes/No/Not sure (Plus evidence)* 4. Is a description of the framework components provided? *Yes/No/Not sure (Plus evidence)*
2. How the framework has been used to develop the evaluation methods and data collection. Are relevant components applied?	5. Is the evaluation stated as explicitly based on the framework components? *Yes/No/Not sure (Plus evidence from the method of how the framework components have been applied to inform evaluation methods & data sources)*
3. How the framework has been applied to the reporting of outcomes.	6. Are the outcome measures discussed in the result/discussion sections linked to the relevant framework components? *Yes/No/Not sure (Plus evidence)* 7. How many of the framework components are linked to data sources/measures? *All the main framework components / At least one, but not all /None of the components are linked to data (Plus evidence)*
4. Reporting use of framework fully.	8. Are any details of adaptations in how the framework has been applied provided? *Yes/No/Not sure (Plus evidence)* 9. Are any details of limitations and strengths in how the framework has been applied or suggestions for how it could be optimised provided? *Yes/No/Not sure (Plus evidence)*

Data extraction was completed by JF and validated by JM. For the data related to study characteristics, a sample of 20% of studies were checked and validated and any disagreements were resolved through discussion. For our checklist of indicators used to appraise the quality of reporting of framework use, we first tested the indicators by independently extracting data for a small sample of papers and discussed any differences to refine the process and reach a consensus in how to apply the indicators to extract data. We then independently validated a sample of 20% of studies and calculated the level of agreement as a percentage in order to validate the data extraction process. Any further disagreements were resolved through discussion. We used narrative synthesis to summarise the use and reporting of frameworks within the included studies.

## Results

**Fig. 1:**
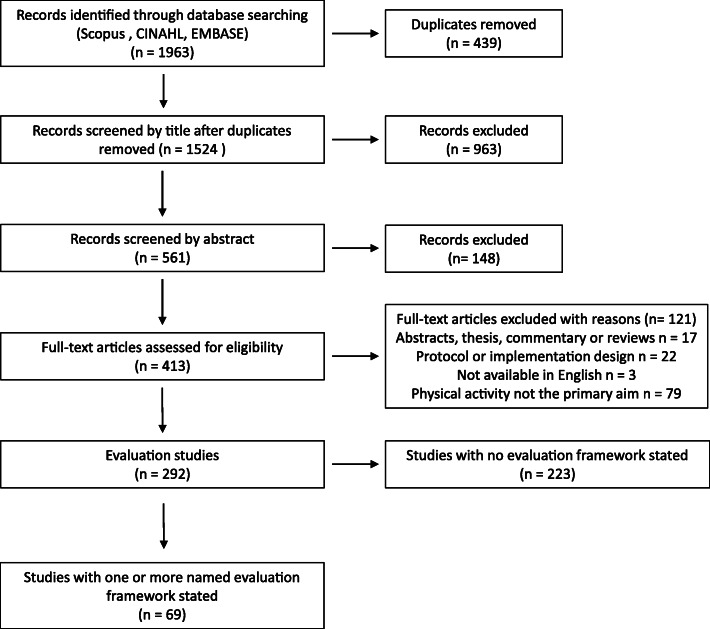
PRISMA diagram of screening process

The search identified 1524 studies once duplicates had been removed. The PRISMA diagram for the screening is shown in Fig. 1. We identified a total of 292 evaluation studies of physical activity interventions. Only 69 (23%) of these mentioned using an evaluation framework. From the reference list of these 69 studies we identified an additional eight companion studies, however none mentioned using an evaluation framework so were not included. Three interventions were reported in more than one of the included studies; therefore the 69 included studies represent 64 different physical activity interventions.

Table 4 summarises the evaluation frameworks which were reported as being used and the number of studies using each framework. A total of 16 different evaluation frameworks were identified. These include frameworks that provide guidance on evaluation specifically, such as the Process Evaluation Plan [30], and frameworks that provide guidance on intervention planning and development but that facilitate evaluation and reporting, such as Precede-Proceed [34], Intervention Mapping [35] and Logic Models [17]. The frameworks most frequently reported were RE-AIM [10], Saunders and Joshi’s process evaluation plan [30] and Steckler and Linnans’ process evaluation guidance for public health [31]. RE-AIM [10] and the MRC guidance for development and evaluation of complex interventions [16] were the frameworks most frequently reported as being used as a single framework to inform the evaluation study. Realist evaluation [33] was only reported in four studies but was in all cases used as a standalone framework rather than in combination with other frameworks. Fourteen studies reported applying more than one framework (Table 6). The frameworks most frequently reported as being used in combination with others were Saunders and Joshi’s [30] and Steckler and Linnan’s [31] process evaluation frameworks. Both these frameworks provide a similar step-wise approach to process evaluation. The MRC guidance on process evaluation [32] and logic models [17] were also reported in several studies, both as a standalone framework and in combination with other frameworks.

**Table 4:** Evaluation frameworks reported within the 69 studies

Named Framework	Number of studies reporting
RE-AIM [10]	27
Developing a process evaluation plan [30]	12
Process evaluation for public health [31]	10
MRC Guidance on evaluation of complex interventions [16]	8
MRC Guidance on process evaluation [32]	8
Logic Model [17]	7
Realist Evaluation [33]	4
Precede-Proceed [34]	3
Intervention Mapping [35]	2
Outcome Model [36]	2
CDC Framework [37]	1
Evaluation: a Systematic Approach [38]	1
Model of Implementation [39]	1
WHO Process Evaluation Workbook [40]	1
Swiss Model for Outcome Classification [41]	1
Concepts in process evaluation [42]	1

Note: 14 papers referred to more than one of these frameworks informing the evaluation

### Study characteristics

Study characteristics are shown in the supplementary material (Additional File 1). The frameworks have been used in a wide range of contexts and circumstances. Most of the criteria used to describe the interventions were clearly specified, and there was good agreement in the sample validated independently. The study population was reported in all studies; 37 studies (54%) reported interventions targeting children or young adults, 24 (35%) targeted adults, and five (7%) targeted older people. The remaining three (4%) studies did not specify an age group but implied the intervention was targeted at multiple population groups or the general public. Relevant details of demographic and/or health status of target populations were also described fully in studies where this was relevant: interventions targeting populations with or at increased risk of diabetes, the metabolic syndrome or heart disease; low socio-economic groups; and women or men only. Details of the included population were reported variously as sample size, participants recruited, or the number of intervention sites. Intervention setting was described in all studies; 28 (40%) were implemented in schools (including pre-schools), 13 (20%) in health care settings, four (6%) in the workplace, and 24 (35%) in other community settings (e.g. youth groups, churches). All studies provided some description of the intervention components (i.e. activities delivered), although the level of detail was variable. For example, most studies described specific activities delivered (e.g. walking, dance, counselling, staff training, online tools), whilst fewer studies provided details of who delivered the intervention, the mode of delivery, the dose, or modifications to the delivery of the intervention. Most studies were multi-component and described several activities delivered together. Training (*n* = 50, 72%), education (*n* = 47, 68%) and enablement (*n* = 42, 61%) were the most frequently reported intervention functions stated in the studies. Studies less frequently reported modelling (*n* = 12, 17%), incentivisation (*n* = 9, 13%), environmental restructuring (n = 9, 13%) and persuasion (n = 4, 6%).

Additional File 1 shows the data we extracted related to the study objectives, study design and outcomes reported. Study designs included quantitative, qualitative and mixed-methods studies, controlled trials, quasi-experimental, case studies and hybrid designs. Thirty-five (51%) studies were described as a process evaluation and 15 (22%) as an outcome evaluation. In addition to physical activity outcomes, a range of secondary outcomes were reported: 52 (75%) reported on various implementation measures e.g. reach, dose, fidelity and maintenance; 14 (20%) reported outcomes related to anthropometric measures; and 15 (22%) reported details of participant demographics. Only nine (13%) studies reported outcome measures related to quality of life and only five (7%) reported on economic or cost analysis.

### Appraisal of the quality of reporting on the use of evaluation frameworks

Table 5 shows the data extracted on the use and reporting of an evaluation framework for studies referring to a single framework, and Table 6 shows the data for studies referring to more than one framework. The level of agreement for the validation of data extracted for these items was 80%. Six studies mentioned a framework but did not state that the evaluation was informed by it. These included one study that provided a logic model but made no reference to this other than in the figure caption [64], and four studies that mentioned the MRC guidance on evaluating complex interventions and one that mentioned the MRC guidance on process evaluation of complex interventions but did not explicitly state that the study was informed by these guidance documents [69–71, 75, 112] (four of these were companion studies relating to the same intervention). In three (4%) further studies the description lacked sufficient clarity to determine whether the study was intended to be based on the reported framework or not; for example these referred to the formulation of a logic model but did not describe the evaluation and outcomes as being based on the logic model [48, 49, 65]. The remaining 60 (87%) studies all stated that the evaluation was informed by one or more specified framework. However, based on the extracted data on how studies had reported framework components, how these had been applied and how the results linked to the framework components, we identified only 51 (74%) of the studies as being explicitly based on the reported framework.

**Table 5:** Appraisal of use and reporting of an evaluation framework in studies using a single evaluation framework

Framework(s)	Intervention name	First author & publication	Framework stated in introduction	Framework components described	Study stated as based on framework	Outcomes linked to components	Framework mentioned	Explicitly based on framework	No. of components linked	Adaptations described	Limitations described
CDC Framework [37]	WAVE	Meng [57]	No	No	Yes	Yes	√		Not sure	No	No
Developing a Process-Evaluation Plan [30]	APAN	Blackford [58]	No	Yes	Yes	Yes		√	All	Yes	Yes
	Exercise Counselling	McCarthy [59]	Yes	Yes	Yes	Yes		√	At least one	No	No
	NECaSP	Curry [60]	No	No	Yes	Not sure		√	Not sure	Yes	Yes
	PACES	Webster [61]	Yes	Yes	Yes	No	√		Not sure	No	No
	ToyBox-study	De Craemer [62]	Yes	Yes	Yes	Yes		√	At least one	Yes	Yes
Evaluation: a Systematic Approach [38]	FLEX	Wright [63]	No	Yes	Yes	Yes		√	Not sure	Yes	No
Logic Model	Girls Active	Harrington [64]	No	No	No	No	√		Not sure	No	No
	GOTR	Ullrich-French [65]	No	No	Not sure	Yes	√		Not sure	Yes	No
	Healthy Start	Chow [66]	No	No	Yes	Not sure	√		At least one	No	Yes
	School–Community Linked PA	Griffiths [67]	No	No	Yes	Yes		√	At least one	No	Yes
Model of Implementation [39]	MAGNET	Burkart [68]	Yes	Yes	Yes	Yes		√	Not sure	No	No
MRC Guidance for Development & Evaluation of Complex Interventions [16]	Action 3.30	Jago [69]	No	No	No	No	√		Not sure	No	No
	BGDP	Jago [70]	No	No	No	Yes	√		At least one	No	No
	BGDP	Sebire [71]	Yes	No	No	No	√		At least one	No	No
	GoActive	Corder [72]	Yes	No	Yes	No	√		Not sure	No	No
	Movement as Medicine	Avery [73]	Yes	Yes	Yes	Yes		√	At least one	No	Yes
	STAND	Biddle [74]	Yes	No	No	No	√		None	No	No
MRC Process Evaluation of Complex Interventions [32]	BGDP	Sebire [75]	Yes	No	Yes	Not sure	√		At least one	Not sure	Yes
	BGDP	Sebire [76]	No	No	No	No	√		None	No	No
	LPAW	Lefler [77]	Yes	No	Yes	No	√		None	No	No
	PACE-UP	Furness [78]	Yes	Yes	Yes	Yes		√	At least one	No	No
	We Act	Bonde [79]	Yes	Yes	Yes	Yes		√	All	No	No
Outcome Model [36]	Healingo Fit	Dadaczynski [80]	No	No	Yes	Yes		√	At least one	No	No
PRECEDE PROCEED [81]	SPACE	Tucker [82]	No	No	Yes	No	√		Not sure	No	No
Process Evaluation for Public Health [31]	Group fitness	Sofija [83]	No	Yes	Yes	Yes		√	All	No	No
	PAC	Matthews [84]	Yes	No	Yes	Yes	√		At least one	No	No
RE-AIM [10]	5-As	Galaviz [85]	No	Yes	Yes	Yes		√	All	No	No
	ACTIVE	Christian [86]	No	No	Yes	Yes		√	All	No	No
	CHAM JAM	Reznik [87]	No	Yes	Yes	Yes		√	All	No	No
	COMMUNICATE	Kamada [4]	No	No	Yes	Yes		√	At least one	No	No
	Enhance®Fitness	Kohn [88]	Yes	No	Yes	Yes		√	At least one	No	No
	Enhance®Fitness	Petrescu-Prahova [89]	Yes	Yes	Yes	Yes		√	At least one	Yes	Yes
	FAN	Wilcox [90]	Yes	No	Yes	Not sure		√	At least one	No	No
	FitEx & ALED	Harden [91]	Yes	Yes	Yes	Yes		√	At least one	No	Yes
	Guided Walking	Baba [92]	Yes	Yes	Yes	Yes		√	All	No	Yes
	HKOS	Economos [93]	Yes	Yes	Yes	Yes		√	All	Yes	No
	Healthy Start-Départ Santé	Ward [94]	Yes	Yes	Yes	Yes		√	All	No	No
	Healthy Together	Jung [95]	Yes	Yes	Yes	Yes		√	All	No	No
	IMIL	Allar [96]	Yes	Yes	Yes	Yes		√	At least one	Yes	Yes
	ManUp	Caperchione [97]	Yes	Yes	Yes	Yes		√	All	No	Yes
	PAFES	Gonzalez-Viana [98]	Yes	Yes	Yes	Yes		√	All	No	Yes
	Promotora Community Health Program	Schwingel [99]	Yes	Yes	Yes	Yes		√	All	Yes	Yes
	RCP & ACP	Paez [100]	Yes	Yes	Yes	Yes		√	All	Yes	Yes
	Sport England funded project	Koorts [101]	Yes	No	Yes	Yes		√	All	Yes	Yes
	Stair Climbing	Bellicha [102]	No	Yes	Yes	Yes		√	At least one	Yes	Yes
	STEPs & LET US Play	Beets [103]	No	No	Yes	Yes		√	At least one	Not sure	Yes
	STEPs & LET US Play	Beets [104]	No	No	Yes	Yes		√	At least one	Yes	Yes
	SAGE	Lee [105]	Yes	Yes	Yes	Yes		√	All	No	Yes
	TAME health	Lewis [106]	Yes	No	Yes	Yes		√	All	No	Yes
	Walking Works	Adams [107]	Yes	Yes	Yes	Yes		√	All	No	Yes
Realist Evaluation [33]	CBHEPA	Herens [108]	Yes	Yes	Yes	Yes		√	All	Not sure	Yes
	Local Authority Sport & PA	Daniels [109]	Yes	Yes	Yes	Yes		√	All	Yes	Yes
	Local Environment Model	Willis [110]	Yes	Yes	Yes	Yes		√	All	No	No
	Project SoL	Mikkelsen [111]	No	Yes	Yes	No	√		Not sure	No	No

**Table 6:** Appraisal of use and reporting of the use of evaluation frameworks in studies using multiple frameworks

No. of Frameworks & references	Intervention Name	First author & publication	Framework stated in introduction	Framework components described	Stated as based on framework	Outcomes linked to components	Framework mentioned	Explicitly based on framework	No. of components linked	Adaptations described	Limitations described
2 [17, 30]	WWPP	Fournier [43]	Yes	No	Yes	Yes		√	At least one	No	No
2 [30, 34]	SPACE	Driediger [44]	Yes	No	Yes	Yes		√	At least one	No	No
2 [30, 31]	IDEFICS	Verloigne [45]	Yes	No	Yes	Not sure	√		Not Sure	No	Yes
2 [30, 31]	PA for grandparents	Young [46]	Yes	Yes	Yes	Yes		√	At least one	No	No
2 [30, 31]	It’s LiFe!	Verwey [47]	No	Yes	Yes	Yes		√	At least one	No	Yes
2 [17, 41]	Classes in Motion	Grillich [48]	No	No	Not sure	Yes	√		At least one	No	No
2 [16, 17]	ENGAGE-HD	Quinn [49]	Yes	No	Not sure	Not sure	√		Not Sure	No	No
2 [10, 16]	Move for Well-being in School	Smedegaard [50]	Yes	Yes	Yes	Yes		√	All	Yes	No
2 [31, 32]	WAVES	Griffin [51]	Yes	No	Yes	Yes		√	At least one	Yes	Yes
2 [31, 40]	Walk Well	Matthews [52]	No	Yes	Yes	Yes		√	At least one	No	No
3 [10, 30, 31]	‘BeweegKuur’	Berendsen [53]	Yes	Yes	Yes	Yes		√	All	Yes	Yes
3 [10, 32, 35]	Workplace intervention for Nurses	Torquati [54]	Yes	No	Yes	Yes		√	All	Yes	Yes
3 [30, 31, 36]	SLIMMER	van Dongen [55]	Yes	Yes	Yes	Yes		√	All	No	Not sure
5 [17, 31, 34, 35, 42]	SHAPES	Saunders [56]	Yes	No	Yes	Yes		√	At least one	No	Yes

Forty-four studies (64%) referred to the framework(s) in the introduction, while thirty-six (52%) provided a description of the framework components. Fifty-three (77%) reported outcomes linked to relevant framework components, the remaining sixteen (23%) studies provided no evidence of how the outcomes reported were linked to the framework components. Only 26 (38%) studies provided detailed descriptions consistently across all of the indicators; this included 13 that used RE-AIM, three that used Realist evaluation, two that used the MRC guidance on process evaluation, and two that used Saunders and Joshi’s process evaluation framework. Four studies [46, 50, 55, 113] that had applied frameworks in combination also consistently reported details of the frameworks and their use across all indicators. Twenty-nine studies (42%) described strengths or limitations, whilst only 17 (25%) described adaptations in how the framework had been used.

## Discussion

### The extent to which evaluation frameworks have been used and reported

This is the first systematic review that has attempted to comprehensively assess the use of evaluation frameworks within evaluations of physical activity interventions. We identified 292 evaluation studies of interventions in which physical activity was the primary goal, published between 2015 and the date of our search. Only 69 (23%) of these studies reported using an evaluation framework; within these 16 different frameworks were mentioned. Given that we previously identified 68 published evaluation frameworks that could be used to facilitate evaluation of physical activity interventions [14], our findings highlight that evaluation frameworks are under-used and/or under-reported. Their limited use suggests missed opportunities to apply frameworks to guide evaluation and reporting in intervention studies. For example, despite recommendations in several guidance documents to use logic models to support intervention development and evaluation [15, 32, 114], logic models were only referred to in seven of the studies, and their application was poorly reported. None of the studies reported using any frameworks that have been developed specifically for use in physical activity programme evaluation such as the SEF for physical activity interventions [15]. This may be explained by its more limited guidance on process evaluation, given that 51% of the studies were a process evaluation and 75% reported implementation measures. The SEF was developed for use in a UK practice context and may therefore be less likely to be used in a research led intervention than a real-world programme evaluation. Its absence from any of the studies in this review suggests not just a limited use made of it but also highlights the gap between research and practice and the challenges of reporting real world evaluations in the scientific literature. The more frequent use and reporting of RE-AIM may be because it provides guidance on both outcome and process evaluation components. However, its use may also be influenced by its greater exposure within the literature.

Framework use, choice of framework and the quality of reporting is likely to be influenced by the intervention’s context and circumstances in which they are used. Many of the studies (*n* = 35, 51%) were process evaluations and it therefore follows that the most frequently reported frameworks were process evaluation frameworks. However, we found that a range of frameworks were used across different intervention types, contexts and study designs. This suggests that many evaluation frameworks are widely applicable and the decision to use and report a framework is more critical than the choice of which framework to use.

### The quality of reporting with regards to how frameworks were used

Our checklist of indicators (Table 3) enabled us to appraise the quality of use and reporting of evaluation frameworks. There was considerable variation in the quality of reporting of framework use (Tables 5 and 6). Whilst some studies did report the framework and how it had been used consistently across all indicators in our checklist, others were less consistent in the quality of reporting and some only mentioned a framework without specifying the details of its use. In some studies, the evaluation was reported as being informed by a framework even where there was little evidence of the evaluation being based on it.

Studies tended to be poorer at describing framework components and adaptations or limitations in how these had been used, whilst links between outcome measures and framework components were more clearly described. For example, those which applied just one or more framework’s components, rather than all the components, provided very little explanation or rationale for these adaptations. Publishing constraints can mean that reporting an evaluation study fully requires companion papers or supplementary files [16]. However, where this was done, we found that there was often inconsistency in reporting the use of frameworks across the different reported elements e.g. [61, 69–71, 75, 76, 79, 115]. More detailed and consistent reporting of the framework components and how these have been applied would help those trying to understand the intervention effectiveness fully.

It is inevitable that some frameworks lend themselves to better quality reporting. For example, studies using RE-AIM and Realist evaluation provided a more consistent report of their use across all indicators. RE-AIM is a structured framework; whilst Realist evaluation is a methodological approach, it too provides a guiding framework to facilitate a systematic evaluation and as such has been referred to as a framework within this paper. Both RE-AIM and Realist evaluation have a clear set of components that are relevant to both process and outcomes; they are therefore applicable to a range of evaluation objectives and can be used to identify appropriate data sources. Many of the studies using RE-AIM provided a full description of the components, an explanation of how these linked to data sources, and used the framework components to structure the reporting of findings. In this way the framework facilitated both a systematic evaluation and consistent reporting. RE-AIM was the most frequently used framework. There is a body of literature on how RE-AIM has been developed and used over time [116], and examples of its application. This may have helped to build a better understanding of how its components are defined and how they can be linked to data sources. Some of the less structured guidance documents, for example the MRC guidance on the development and evaluation of complex interventions [16], were used more loosely as a framework, particularly in studies that used more than one framework in combination. This does not necessarily equate to a poorer quality evaluation. However, we suggest those studies drawing on several frameworks and general guidance documents would benefit from a more detailed reporting of how these have been used to assist the reader in understanding which intervention components are reported on, and why. Whilst there is variability in the quality of reporting of how frameworks have been used, this review does highlight that evaluation frameworks can, when used appropriately, facilitate a systematic evaluation, and that studies that use a framework can facilitate systematic reporting of the evaluation process and outcomes.

Despite recommendations on the importance of fully reporting contextual factors and intervention components, and guidance within the frameworks to facilitate this [16, 32], our review supports previous review findings [23, 26] that the reporting of intervention components is variable, with wider effects (e.g. quality of life and costs) and wider contextual factors (e.g. dose, intervention modifications) being particularly poorly reported. The Behaviour Change Wheel was developed to characterise intervention types and identify behaviour change techniques as “active ingredients” to improve the reporting and synthesis of evidence of what works in different populations and settings [21]. Yet we found ambiguity in the way in which studies reported intervention functions. It is noteworthy that intervention function was the item where we initially had most disagreement in the data extraction validation process and we would argue that clearer specification, or mapping of intervention functions against behaviour change techniques, would make them more useful in characterising interventions. Poor reporting of intervention components and types limits their comparability and transferability.

If evaluation studies are to contribute to an evidence base on which policymakers, practitioners and researchers can draw to inform the development and implementation of interventions, both the framework and intervention components need to be more clearly defined and documented. Clear, consistent and full reporting of interventions and their evaluation is essential to ensure that critical evidence gets shared and used to develop understanding of causal mechanisms, contextual factors and good practice [25]. This is vital to allow resources and efforts to address public health issues, such as increasing physical activity, to be focused on effective and efficient intervention components.

Where frameworks are used, their application to guide the full evaluation process from planning to reporting can improve the quality of reporting of their use. A focus on evaluation at the design and development stages of interventions and a clear understanding of the purpose of the evaluation can help to ensure outcome measures are linked to framework components. However, there is a need to improve understanding of how framework and intervention components are defined. Training and documentation can play a role, but more consistent and precise reporting within the scientific literature is needed. Our set of indicators (see Table 3) can be used to guide the reporting of framework use. Those reporting an evaluation study can apply the indicators as a checklist to provide a clear and consistent description of how framework components have been applied across all stages of the evaluation. Reviewers and journal editors can also play a role in using the checklists available to appraise evaluation reports.

### Strengths and limitations

The strengths of this study are that we developed a comprehensive checklist of indicators to appraise the use and reporting of evaluation frameworks, based on a widely accepted coding scheme designed to assess the use and reporting of theory [29]. Our checklist and its use as a guide to data extraction was piloted and developed iteratively, and agreed by all authors. This enabled us to review the use and reporting of different frameworks.

Limitations of our study include the fact that some studies may use frameworks or framework components in a way that is implied but not explicitly stated, and we acknowledge that this may have led to underrepresentation of the full use made of evaluation frameworks. A more detailed assessment of evaluation studies against each specific framework’s components may have provided greater insight into the limitations or fidelity of use and reporting of frameworks. This was not practical to do within a single review of multiple evaluation frameworks. Extracting details of outcome measures (findings) and intervention characteristics for all physical activity evaluation studies may have enabled a fuller appraisal of the quality of the studies and a comparison between those using and those not using an evaluation framework. This may have provided further insights on the impact of using evaluation frameworks on the quality of the evaluation study, however this was beyond the scope of this review.

## Conclusion

Despite the use of evaluation frameworks being advocated to improve the rigour of evaluation studies, frameworks are underused and reported inconsistently in many studies. Applying an evaluation framework to inform both the evaluation and reporting of physical activity intervention studies facilitates a more systematic evaluation study. However, intervention and framework components need to be more precisely and consistently defined and documented to help improve the quality of reporting. Variability in the quality of reporting limits the comparability and transferability of evidence. This means that critical evidence that could be used to inform interventions to support the health of the population is not making it into the public domain. The indicators we developed enabled us to appraise the use and reporting of a range of different evaluation frameworks within evaluations of physical activity interventions. These indicators can be used by those reporting an evaluation to guide them in developing a systematic evaluation report, and by reviewers and journal editors to appraise evaluation studies that have reported the use of an evaluation framework.

## Supplementary information

**Additional file 1.** Study Characteristics. A summary of data extracted related to study characteristics to assess the context and circumstances in which evaluation frameworks have been used.

## Data Availability

The dataset(s) supporting the conclusions of this article are included within the article (and it’s additional files).

## Abbreviations

ACTIVE: Active Children through Incentive Vouchers – Evaluation
ALED: Active Living Every Day
APAN: Albany Physical Activity and Nutrition
5-As: Assess, Advise, Agree, Assist, Arrange for physical activity counselling
BCT: Behaviour Change Technique
BGDP: Bristol Girls Dance Project
CBHEPA: Dutch Community-Based Health-Enhancing Activity Programs
CDC: Center for Disease Control and Prevention
CHAM JAM: Children’s Hospital at Montefiore Joining Academics and Movement
CoM: Communities on the Move
COMMUNICATE: COMMUNIty-wide Campaign To promote Exercise
FAN: Faith, Activity, and Nutrition
FitEx: Fit Extension
FLEX: Fuelling Learning through Exercise
GOTR: Girls On The Run
Healingo Fit: Health Integrated Gaming Online
HKOS: Healthy Kids Out of School
HT: Healthy Together
IDEFICS: Identification & prevention of Dietary & lifestyle-induced health EFfects In Children and infants
IMIL: I am Moving, I am Learning
LPAW: Lifestyle Physical Activity for Older Women
MAGNET: Mothers And dauGhters daNcing togEther Trial
MRC: Medical Research Council
NECaSP: Newham’s Every Child a Sports Person
PA: Physical Activity
PAC: Physical Activity Consultation
PACES: Partnerships for Active Children in Elementary Schools
PACE-UP: Primary care pedometer based walking trial
PAFES: Physical Activity, Sports, and Health Plan
RCP & ACP: Recreovia program & Academia da Cidade program
RE-AIM: Reach, Efficacy, Adoption, Implementation, Maintenance
SAGE: Sustainability via Active Garden Education
SEF: Standard Evaluation Framework
SHAPES: Study of Health and Activity in Preschool Environments
SLIMMER (SLIM): iMplementation Experience Region Noord- en Oost-Gelderland
SPACE: Supporting Physical Activity in the Childcare Environment
STAND: Sedentary Time ANd Diabetes
STEPs: Strategies To Enhance Practice in YMCA-operated After School Programmes
STROBE: Statement for Reporting Observational studies in Epidemiology
TAME health: Testing Activity Monitors’ Effect on health
TIDieR: Template for Intervention Description and Replication
WAVE: WAVE~Ripples for Change
WWPP: Walking with Poles Program
WAVES: West Midlands ActiVe lifestyle and healthy Eating in School children
WHO: World Health Organization
